# Early glycaemic exposure and cancer risk in people with newly diagnosed type 2 diabetes

**DOI:** 10.1007/s00125-026-06758-7

**Published:** 2026-06-05

**Authors:** Xinge Zhang, Aimin Yang, Hongjiang Wu, Eric S. H. Lau, Mai Shi, Baoqi Fan, Elaine Chow, Alice P. S. Kong, Juliana C. N. Chan, Ronald C. W. Ma, Andrea O. Y. Luk

**Affiliations:** 1 Department of Medicine and Therapeutics, The Chinese University of Hong Kong, Hong Kong, Special Administrative Region China; 2 Hong Kong Institute of Diabetes and Obesity, The Chinese University of Hong Kong, Hong Kong, Special Administrative Region China; 3 Li Ka Shing Institute of Health Sciences, The Chinese University of Hong Kong, Hong Kong, Special Administrative Region China

**Keywords:** Cancer, Glycaemic exposure, HbA_1c_, Legacy effect, Type 2 diabetes

## Abstract

**Aims/hypothesis:**

The aim of this study was to determine whether overall and time-specific patterns of hyperglycaemia, particularly soon after diagnosis, are associated with incident cancer in adults with newly diagnosed type 2 diabetes.

**Methods:**

We retrospectively analysed a territory-wide cohort of 52,926 Hong Kong Chinese people with newly diagnosed type 2 diabetes. We examined cancer risk across groups of individuals classified according to their time-weighted mean HbA_1c_ over the entire follow-up period (*n*=49,978) or during specific early exposure periods (*n*=39,185). A weighted cumulative exposure model was used to determine the role of historical HbA_1c_ exposures in cancer development (*n*=49,966).

**Results:**

Among 49,978 individuals with newly diagnosed type 2 diabetes, 1758 cancer events occurred. Each 11 mmol/mol (1%) increase in time-weighted mean HbA_1c_ was associated with a 27% relative higher risk of cancer at any site (HR 1.27; 95% CI 1.20, 1.33). Within the first 2 years after diagnosis, a time-weighted mean HbA_1c_ ≥53 mmol/mol (≥7.0%) vs <53 mmol/mol (<7.0%) was associated with a 30–75% relative higher risk of cancer at any site, depending on the specific HbA_1c_ category, even after adjusting for subsequent HbA_1c_. Longer durations of early exposure were associated with higher risk, reaching 51–213% in the first 5 years of exposure. Earlier high HbA_1c_ exposures contributed more strongly to cancer risk than later exposures. A 11 mmol/mol (1%) HbA_1c_ reduction at 1–2 years was associated with a 6% relative lower cancer risk over a hypothetical 10 year window (HR 0.94; 95% CI 0.91, 0.98), whereas reductions after 5 years showed no significant risk differences.

**Conclusions/interpretation:**

Overall, hyperglycaemic exposure was associated with an elevated long-term cancer risk in type 2 diabetes. Notably, individuals who showed better glycaemic management soon after diagnosis exhibited a lower cancer risk than those whose glycaemic management improved later, despite comparable overall glycaemic burdens.

**Graphical Abstract:**

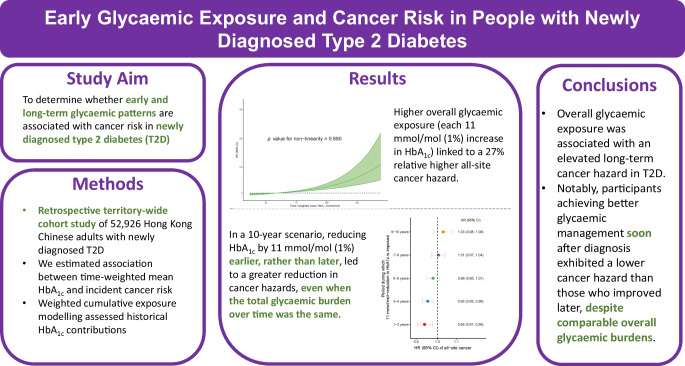

**Supplementary Information:**

The online version contains peer-reviewed but unedited supplementary material available at 10.1007/s00125-026-06758-7.


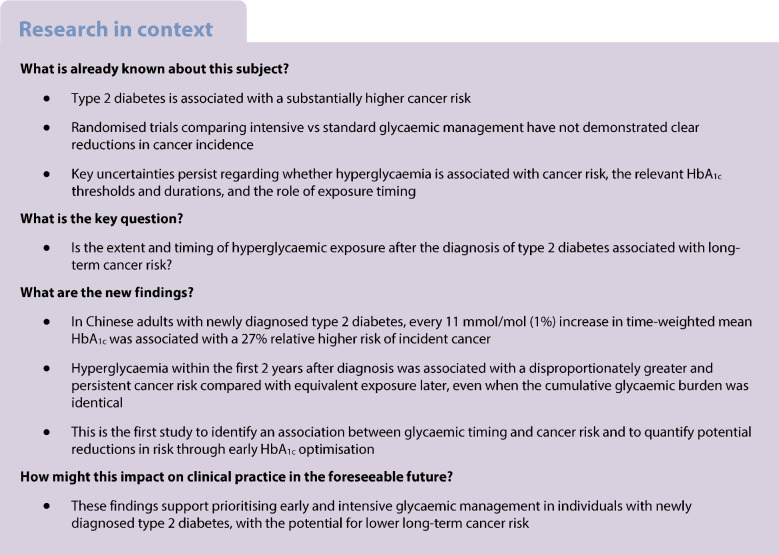


## Introduction

Diabetes and cancer are two non-communicable diseases of significant global health concern. Approximately 537 million adults worldwide, or one in ten, were estimated to be living with diabetes in 2021 [1]. Meanwhile, cancer accounts for over 10 million deaths annually worldwide [2]. Cancer is emerging as a leading cause of death among individuals with diabetes, partly due to reduced deaths from cardiovascular disease and increased lifespan [3]. Type 2 diabetes is associated with an increased risk of most cancers [4–6], potentially driven by hyperinsulinaemia and chronic hyperglycaemia [4]. Several observational studies have linked higher fasting glucose and HbA_1c_ to elevated cancer risk, independent of BMI [7, 8]. However, randomised controlled trials have failed to show a reduction in cancer incidence as a result of intensive glycaemic management vs standard care [9], potentially due to modest between-group HbA_1c_ differences, sample size limitations, and other methodological issues [10]. The extent to which hyperglycaemia exposures may contribute to incident cancer in people with type 2 diabetes remains uncertain. This uncertainty also raises the question of whether glycaemic exposure is associated with a cumulative and lasting pattern of cancer risk, similar to that seen for vascular complications [11]. Importantly, it is not known whether hyperglycaemic exposures early in the disease course are associated with different oncogenic potential compared with later exposures.

In this territory-wide study of Hong Kong Chinese adults with newly diagnosed type 2 diabetes, we aimed to (1) assess the association between the overall glycaemic burden (expressed as time-weighted-mean HbA_1c_ during the follow-up period) and cancer risk; (2) examine how cancer risk changes with the intensity and duration of hyperglycaemia after diabetes diagnosis, independent of subsequent glycaemic fluctuations; and (3) quantify the effect of early vs late glycaemic exposure, if any, on cancer risk by modelling the contribution of historical HbA_1c_ exposure to future cancer risk.

## Methods

### Overview

**Fig. 1:**
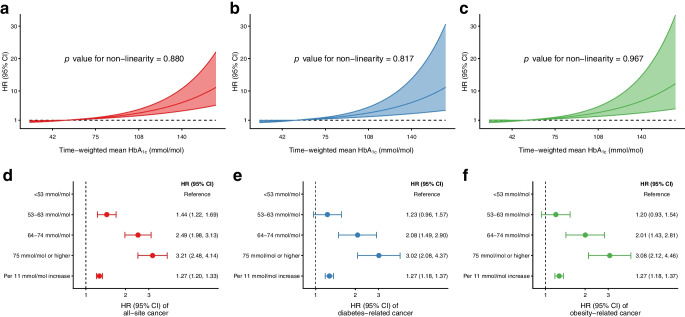
Dose–response relationship between time-weighted mean HbA_1c_ during the entire observation period and incident risks of cancer at any site (**a**), diabetes-related cancer (**b**) and obesity-related cancer (**c**), with corresponding forest plots (**d**–**f**). The *x-*axis of the forest plots is presented on a logarithmic scale. Models were adjusted for age at diagnosis, sex, calendar year at diagnosis, smoking, drinking, systolic and diastolic BP, BMI, triglyceride-to-HDL-cholesterol ratio, LDL-cholesterol, history of CVD, congestive heart failure or chronic kidney disease at baseline, and use of insulin or oral glucose-lowering drugs (metformin, sulfonylureas, DPP-4 inhibitors, thiazolidinediones and others) during the observation period

A detailed flow chart of the study design is shown in electronic supplementary material (ESM) Fig. 1. For aim 1, the date of diagnosis of diabetes was considered the baseline. Individuals were followed-up until death, 31 December 2019, or 2 years before cancer onset, whichever came first. This 2 year lag was applied to reduce reverse causality, as undiagnosed cancer may alter glycaemic control before clinical detection. This ensures that HbA_1c_ reflects usual status rather than effects of occult malignancy, based on prior evidence showing that 2 years sufficiently minimises such confounding [12]. We compared cancer risks across glycaemic burden groups over the entire observation period. For aim 2, we adapted an approach used in a previous study that examined early glycaemic exposure and cardiovascular complications [11]. Specifically, we calculated time-weighted mean HbA_1c_ within five defined early periods: 0–1, 0–2, 0–3, 0–4 and 0–5 years. We then assessed subsequent cancer risk across groups defined by HbA_1c_ levels within each period. The end of each early exposure period was considered the baseline. For aim 3, the data of diagnosis of diabetes was considered the baseline. We divided the follow-up period into 2 year intervals and applied a weighted cumulative exposure (WCE) model [13, 14], assigning unequal weights to historical HbA_1c_ to account for varying risk contributions over time. We reported the study according to the recommendations of the STROBE checklist for cohort studies (Strengthening the Reporting of Observational Studies in Epidemiology).

### Study population

Hong Kong Hospital Authority is a statutory organisation and provides medical care for 90% of the residents of Hong Kong. Between 2000 and 2019, 581,811 individuals, representing 60% of people with diagnosed diabetes attending the Hospital Authority, were enrolled in the authority’s Risk Assessment and Management Programme for Diabetes Mellitus [15], which included detailed assessment of metabolic risk factors and diabetes-related complications. The individuals who participated in the study are largely representative of individuals with diabetes attending the Hospital Authority with respect to sex and age. We do not have information on whether they are similar in terms of regional and socioeconomic factors. For this study, we included people aged 20 years or older with newly diagnosed type 2 diabetes. After excluding those with pre-existing cancer or limited data, and accounting for potential reverse causality (i.e. undiagnosed preclinical cancer influencing glycaemic management rather than vice versa), the final analyses included 49,978, 39,185 and 49,966 individuals for aims 1, 2 and 3, respectively. ESM Methods and ESM Fig. 1 present the detailed exclusion process. The use of anonymised data for this study has received approval from the Joint Chinese University of Hong Kong–New Territories East Cluster Clinical Research Ethics Committee, and the requirement for informed consent was waived due to the retrospective nature of the cohort study.

### Exposures

For aim 1, time-weighted mean HbA_1c_ values were calculated from date of diabetes diagnosis until the end of follow-up. For aim 2, time-weighted mean HbA_1c_ values were calculated for the five early exposure periods. Individuals were grouped according to their mean HbA_1c_: <53 mmol/mol (<7.0%), 53–63 mmol/mol (7.0–7.9%), 64–74 mmol/mol (8.0–8.9%) and ≥75 mmol/mol (≥9.0%). We also calculated time-weighted mean HbA_1c_ values for the subsequent follow-up period after each early exposure window. For aim 3, HbA_1c_ values were assessed at 2 year intervals throughout the follow-up period.

### Covariates

Covariates considered in all analyses included age, calendar year, sex, self-reported smoking status (current, former, never smoker), self-reported alcohol use (current, former, never drinker), and history of cardiovascular disease, heart failure and chronic kidney disease at diagnosis as obtained from medical records. Information on race or ethnicity was not available. Sex was considered in the study design, and was included as a covariate in the analyses. However, no formal sex-specific analysis was conducted. Cardiovascular disease was defined as a history of coronary heart disease, haemorrhagic stroke, ischaemic stroke and/or peripheral vascular disease. Chronic kidney disease was defined as a single recorded eGFR <60 ml/min per 1.73 m^2^ and/or end-stage kidney disease, with end-stage kidney disease being further defined as an eGFR <15 ml/min per 1.73 m^2^, receipt of dialysis, or a history of kidney transplant. Systolic and diastolic BP, BMI, triglyceride-to-HDL ratio and LDL-cholesterol values obtained at diagnosis were included for aims 1 and 2, and repeated measurements of these variables were included for aim 3. Use of insulin therapy and non-insulin glucose-lowering drugs during the follow-up period (aim 1), during early periods (aim 2) and at 2 year intervals (aim 3) was also considered. Non-insulin glucose-lowering drugs were classified into five categories: metformin, sulfonylureas, dipeptidyl peptidase 4 (DPP-4) inhibitors, thiazolidinediones and others.

### Outcome ascertainment

Incident cancers at any site and cancer-related death were identified using in-patient ICD-9 codes 140–208 and ICD-10 codes C00–C97. Diabetes-related and obesity-related cancers were also defined based on prior evidence linking specific cancers to diabetes and obesity, respectively [6, 16, 17]. Detailed descriptions are presented in ESM Table 1.

### Statistical analysis

We summarised participant characteristics stratified by time-weighted mean HbA_1c_ over the entire follow-up period, using means and SD, medians and IQR, or counts (percentages) for continuous or categorical variables, as appropriate.

For aim 1, Fine–Gray models accounting for non-cancer death as a competing event were used to estimate sub-distribution HRs and 95% CIs for cancer risk across HbA_1c_ categories (reference group: <53 mmol/mol [<7.0%]) and for each 11 mmol/mol (1%) increase in continuous HbA_1c_, adjusted for all covariates. Dose–response relationships were assessed using restricted cubic splines with three knots at the 25th, 50th and 75th percentiles of time-weighted mean HbA_1c_.

For aim 2, we used the same competing risk regression approach to estimate HR with 95% CIs across HbA_1c_ categories during each early exposure period (reference: <53 mmol/mol [<7.0%]) for subsequent cancer outcomes, adjusted for all covariates and time-weighted HbA_1c_ during subsequent observation periods. Changes in HR (95% CI) associated with each time-weighted mean HbA_1c_ group in various early exposure periods may reflect patterns related to longer durations of early glycaemic exposure.

For aim 3, laboratory measurements were obtained at 2 year intervals using multiple imputation for missing data. Details are given in ESM Methods. Missing rates were randomly distributed across HbA_1c_ groups in the first 2 years after type 2 diabetes diagnosis (ESM Table 2). We applied WCE models to estimate a weight function, quantifying the contribution of historical glycaemic exposures at various time points after type 2 diabetes diagnosis to subsequent cancer risk. Based on this, we compared cancer risks across several theoretical HbA_1c_ profiles, estimating HRs and 95% CIs. Model performance was evaluated by comparing log-likelihood statistics and AUC at 10 years after type 2 diabetes diagnosis between the WCE and conventional time-varying Cox models. Sensitivity analyses included a negative control outcome (gastrointestinal bleeding) and a positive control outcome (myocardial infarction) [18]. Details of the WCE model are given in ESM Methods.

To further address potential reverse causality bias when estimating the associations of early high HbA_1c_ exposure, whereby subclinical cancer may influence glycaemic management at the time of or shortly after diabetes diagnosis, we conducted a sensitivity analysis by repeating the analysis for aims 2 and 3, extending the exclusion period for incident cancer cases after the type 2 diabetes diagnosis from 2 years to 5 years.

A two-sided *p* value <0.05 was regarded as statistically significant. All analyses were conducted using R software, version 4.3.2 (R Foundation for Statistical Computing, Vienna, Austria).

## Results

### Clinical characteristics

Of the 49,978 individuals included for aim 1, 21,321 (43%), 17,587 (35%), 6689 (13%) and 4381 (9%) had a time-weighted mean HbA_1c_ over the entire follow-up period <53 mmol/mol (<7.0%), 53–63 mmol/mol (7.0–7.9%), 64–74 mmol/mol (8.0–8.9%), and ≥75 mmol/mol (≥9.0%), respectively (Table 1). Overall comparisons across the four groups showed that individuals exposed to higher HbA_1c_ levels were younger, predominantly men, and more likely to be current smokers and drink alcohol regularly. They had a lower BMI and systolic BP but higher triglyceride-to-HDL-cholesterol ratio and LDL-cholesterol, and a higher frequency of use of insulin, metformin, sulfonylureas, DPP-4 inhibitors, thiazolidinediones and other oral glucose-lowering drugs during the observation period.

**Table 1:** Clinical characteristics of individuals at diabetes diagnosis, stratified by time-weighted mean HbA_1c_ during the entire observation period

	Overall	<53 mmol/mol (<7.0%)	53–63 mmol/mol (7.0–7.9%)	64–74 mmol/mol (8.0–8.9%)	≥75 mmol/mol (≥9.0%)	*p* value^a^
Number of individuals	49,978	21,321	17,587	6689	4381	
Number of HbA_1c_ tests	11.0 (6.0–17.0)	11.0 (6.0–15.0)	14.0 (8.0–19.0)	11.0 (6.0–20.0)	5.0 (3.0–13.0)	<0.001
Follow-up duration (years)	6.1 (2.8–9.2)	6.3 (3.3–9.1)	6.5 (3.5–9.4)	5.3 (2.3–8.8)	2.8 (1.0–7.6)	<0.001
Age at diabetes diagnosis (years)	57.0 (50.0–65.0)	60.0 (52.0–67.0)	57.0 (50.0–64.0)	55.0 (47.0–62.0)	54.0 (46.0–61.0)	<0.001
Men	27,149 (54.3)	10,737 (50.4)	9780 (55.6)	3922 (58.6)	2710 (61.9)	<0.001
Smoking status^b^						<0.001
Current smoker	7438 (17.1)	2268 (12.3)	2766 (18.3)	1339 (22.6)	1065 (26.5)	
Previous smoker	6768 (15.6)	2876 (15.6)	2319 (15.3)	941 (15.9)	632 (15.7)	
Never smoker	29,312 (67.4)	13,292 (72.1)	10065 (66.4)	3638 (61.5)	2317 (57.7)	
Alcohol consumption^b^						<0.001
Current drinker	10,369 (24.1)	4189 (22.9)	3646 (24.3)	1509 (25.8)	1025 (25.8)	
Previous drinker	3564 (8.3)	1352 (7.4)	1251 (8.3)	557 (9.5)	404 (10.2)	
Never drinker	29,153 (67.7)	12,731 (69.7)	10,086 (67.3)	3791 (64.7)	2545 (64.0)	
Systolic BP (mmHg)	131.0 (120.0–143.0)	132.0 (121.0–144.0)	131.0 (120.0–143.0)	130.0 (119.0–143.0)	128.0 (117.0–140.0)	<0.001
Diastolic BP (mmHg)	77.0 (70.0–84.0)	77.0 (70.0–84.0)	78.0 (71.0–84.0)	78.0 (71.0–85.0)	77.0 (70.0–84.0)	<0.001
BMI (kg/m^2^)	25.5 (23.1–28.3)	25.8 (23.5–28.4)	25.5 (23.1–28.3)	25.3 (22.8–28.1)	24.8 (22.3–27.8)	<0.001
Triglyceride-to-HDL-cholesterol ratio	1.2 (0.8–1.9)	1.2 (0.8–1.8)	1.2 (0.8–2.0)	1.3 (0.8–2.1)	1.3 (0.8–2.1)	<0.001
LDL-cholesterol (mmol/l)	3.0 (2.4–3.6)	2.9 (2.3–3.6)	3.1 (2.4–3.7)	3.0 (2.4-3.7)	3.0 (2.4-3.7)	<0.001
History of CVD	2661 (5.3)	1344 (6.3)	781 (4.4)	331 (4.9)	205 (4.7)	<0.001
History of chronic kidney disease	2194 (4.4)	1242 (5.8)	634 (3.6)	198 (3.0)	120 (2.7)	<0.001
History of heart failure	310 (0.6)	142 (0.7)	94 (0.5)	43 (0.6)	31 (0.7)	0.407
Use of glucose-lowering drugs during the entire follow-up period						
Insulin	2697 (5.4)	460 (2.2)	816 (4.6)	640 (9.6)	781 (17.8)	<0.001
Metformin	40,840 (81.7)	14,754 (69.2)	16,012 (91.0)	6127 (91.6)	3947 (90.1)	<0.001
Sulfonylureas	21,748 (43.5)	4509 (21.1)	10,049 (57.1)	4466 (66.8)	2724 (62.2)	<0.001
DPP-4 inhibitors	3826 (7.7)	335 (1.6)	1717 (9.8)	1193 (17.8)	581 (13.3)	<0.001
Thiazolidinediones	3032 (6.1)	259 (1.2)	1422 (8.1)	872 (13.0)	479 (10.9)	<0.001
Others	1315 (2.6)	120 (0.6)	466 (2.6)	434 (6.5)	295 (6.7)	<0.001

Values are *n* (%) for categorical variables, and median (IQR) for continuous variables

^a^*p* values for comparisons across the four groups were calculated using the χ^2^ test or Fisher’s exact test for categorical variables and the Kruskal–Wallis test for continuous variables

^b^Percentages for smoking status and alcohol consumption were calculated among participants with non-missing data, which comprised 73.5% and 79.1% of the whole sample, respectively

### Overall glycaemic burden and incident cancer

Over a median follow-up period of 6.1 years (IQR 2.8–9.2), 1758 cancer events were recorded. The three most common incident cancer types were lung cancer (*n*=151), colorectal cancer (*n*=118) and liver cancer (*n*=100). The incidence rates per 10,000 person-years increased from 4.6 (95% CI 4.3, 5.0) to 7.5 (95% CI 6.5, 8.7) for the group with time-weighted mean HbA_1c_ <53 mmol/mol (<7.0%) compared to those with time-weighted mean HbA_1c_ ≥75 mmol/mol (≥9.0%). Compared with an HbA_1c_ <53 mmol/mol (<7.0%), higher HbA_1c_ categories were associated with relative higher risks of incident cancer at any site after multivariable adjustment, with HRs of 1.44 (95% CI 1.22, 1.69) for HbA_1c_ 53–63 mmol/mol (7.0–7.9%), 2.49 (95% CI 1.98, 3.13) for HbA_1c_ 64–74 mmol/mol (8.0–8.9%) and 3.21 (95% CI 2.48, 4.14) for HbA_1c_ ≥75 mmol/mol (≥9.0%). Each 11 mmol/mol (1%) increase in time-weighted mean HbA_1c_ was associated with a 27% relative higher risk of cancer at any site (HR 1.27; 95% CI 1.20, 1.33). Similar patterns of association were observed for incident diabetes- and obesity- related cancers (Fig. 1).

### Early glycaemic exposure and incident cancer

**Fig. 2:**
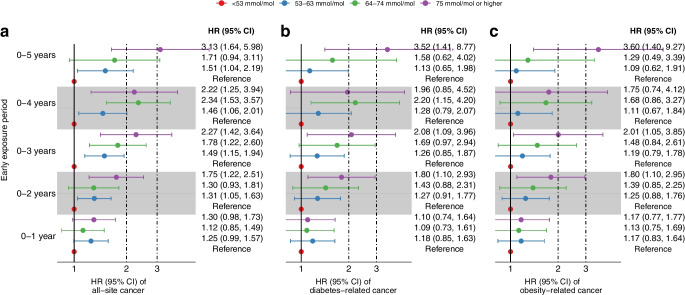
Forest plots for the association between attained HbA_1c_ during each early exposure period and incident risks of cancer at any site (**a**), diabetes-related cancer (**b**) and obesity-related cancer (**c**), with HbA_1c_ <53 mmol/mol (7%) as the reference group. The *x*-axis of the forest plots is presented on a logarithmic scale. Models were adjusted for age at diagnosis, sex, calendar year at diagnosis, smoking, drinking, systolic and diastolic BP, BMI, triglyceride-to-HDL-cholesterol ratio, LDL-cholesterol, history of CVD, congestive heart failure or chronic kidney disease at baseline, and use of insulin or oral glucose-lowering drugs (metformin, sulfonylureas, DPP-4 inhibitors, thiazolidinediones and others), and time-weighted mean HbA_1c_ values during the subsequent observation period

The median follow-up duration, measured from the end of each early exposure period, decreased from 4.9 years (IQR 0.3–13.8) after 0–1 years of early exposure, to 3.6 years (IQR 0.3–11.2) years after 0–5 years of early exposure. An increased hazard of cancer at any site was observed for a time-weighted mean HbA_1c_ ≥53 mmol/mol (7.0%) during 0–2 years and onwards, adjusted for clinical covariates and time-weighted mean HbA_1c_ during follow-up. In 0–2 years following diagnosis, the adjusted HRs (95% CI) were 1.31 (1.05, 1.63), 1.30 (0.93, 1.81) and 1.75 (1.22, 2.51) for HbA_1c_ of 53–63 mmol/mol (7.0–7.9%), 64–74 mmol/mol (8.0–8.9%) and ≥75 mmol/mol (≥9.0%), respectively. The relative hazard of cancer associated with high HbA_1c_ categories further increased as early exposure time lengthened. The adjusted HRs (95% CI) were 1.51 (1.04, 2.19), 1.71 (0.94, 3.11) and 3.13 (1.64, 5.98) for time-weighted mean HbA_1c_ of 53–63 mmol/mol (7.0–7.9%), 64–74 mmol/mol (8.0–8.9%) and ≥75 mmol/mol (≥9.0%) during 0–5 years from diagnosis, respectively. Similar patterns were observed for incident diabetes- and obesity-related cancers (Fig. 2), although not all estimates reached statistical significance. When the cancer exclusion period was extended from 2 to 5 years to reduce reverse causality bias, the confidence intervals for the association between early high HbA_1c_ exposure and cancer risk widened due to the reduced number of events, but the pattern of increasing cancer risk with longer early high HbA_1c_ exposure remained (ESM Fig. 2a).

### Modelling the risk contribution of historical glycaemic exposure

**Fig. 3:**
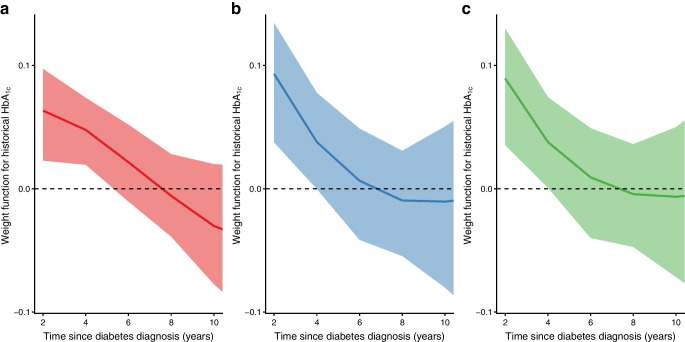
Weight functions for historical HbA_1c_ values for incident risks of cancer at any site (**a**), diabetes-related cancer (**b**) and obesity-related cancer (**c**). A higher weight function means a greater contribution of historical exposure at that time point to the outcome risk. Models were adjusted for age at diabetes diagnosis, sex, calendar year at diagnosis, smoking, drinking, systolic and diastolic BP, BMI, triglyceride-to-HDL-cholesterol ratio, LDL-cholesterol, history of CVD, congestive heart failure and chronic kidney disease, and use of insulin or oral glucose-lowering drugs (metformin, sulfonylureas, DPP-4 inhibitors, thiazolidinediones and others) at baseline

The weight functions estimated from the WCE model suggested that earlier high HbA_1c_ exposure had a greater contribution to cancer risk, with the highest weight observed in the earlier years following diabetes diagnosis (Fig. 3).

**Fig. 4:**
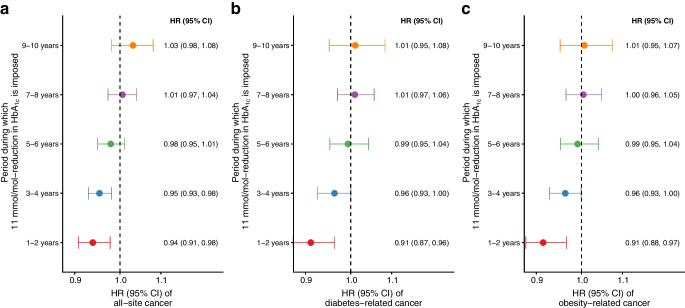
HRs for incident risks for cancer at any site (**a**), diabetes-related cancer (**b**) and obesity-related cancer (**c**) after an 11 mmol/mol (1%) HbA_1c_ reduction implemented at various periods over a 10 year window after type 2 diabetes diagnosis. Models were adjusted for age at diabetes diagnosis, sex, calendar year at diagnosis, smoking, drinking, systolic and diastolic BP, BMI, triglyceride-to-HDL-cholesterol ratio, LDL-cholesterol, history of CVD, congestive heart failure and chronic kidney disease, and use of insulin or oral glucose-lowering drugs (metformin, sulfonylureas, DPP 4 inhibitors, thiazolidinediones and others) at baseline

Figure 4 presents the HRs for incident cancer outcomes associated with several hypothetical HbA_1c_ profiles with comparable overall glycaemic burden. Within a hypothetical 10 year window, achieving a 11 mmol/mol (1%) reduction in HbA_1c_ during specific time periods, while maintaining glycaemic levels in other periods, corresponded to a 6% relative lower risk for cancer at any site (HR 0.94; 95% CI 0.91, 0.98) when the reduction occurred 1–2 years after diagnosis, and a 5% relative reduction in risk (HR 0.95; 95% CI 0.93, 0.98) when the reduction was achieved 3–4 years after diagnosis. However, no significant differences in risk were observed when the reduction was applied in later periods (5–10 years). Similar results were observed for diabetes- and obesity-related cancers.

Compared with the conventional Cox model, which treated HbA_1c_ as a time-varying covariate, the WCE model demonstrated superior predictive performance for long-term cancer risk. This was evidenced by significantly higher log-likelihood statistics and a 15.1–16.2% increase in the 10 year post-diagnosis AUC (ESM Fig. 3). Positive control analysis indicated that early high HbA_1c_ exposures contributed most significantly to long-term myocardial infarction risk, while the association for later exposures diminished over time, consistent with prior evidence [19]. Negative control analyses showed no association between glycaemic exposures and gastrointestinal bleeding risk (ESM Figs 4 and 5). Extending the cancer exclusion period from 2 to 5 years did not alter the overall weighting pattern or the magnitude of the reduction in cancer risk associated with the hypothetical HbA_1c_ reduction (ESM Fig. 2b, c).

## Discussion

In this population-based study of Chinese adults with newly diagnosed type 2 diabetes, we report the following observations: (1) each 11 mmol/mol (1%) increase in time-weighted mean HbA_1c_ values was associated with a 27% relative increase in the risk of cancer at any site, independent of obesity, other metabolic biomarkers and use of insulin therapy; (2) increased glycaemic burden in the first 2 years after diagnosis was associated with increasing cancer risk, and this increased further in those with increased length of exposure to hyperglycaemia up to 5 years from diagnosis; and (3) earlier glycaemic exposures contributed more to cancer risk compared with later exposures. These findings suggest that early and sustained reduction of blood glucose could be relevant in managing the long-term risk of cancer among people with type 2 diabetes.

The relative contributions of hyperinsulinaemia and hyperglycaemia to cancer risk in diabetes remain unresolved. Hyperinsulinaemia, driven by obesity and insulin resistance, may promote tumorigenesis via insulin or IGF-1 receptors [20]. This is supported by a study from the USA (*N*=159,033), in which cancer risk was found to peak alongside C-peptide elevation early in type 2 diabetes [17]. Another study from the UK (*N*=130,764) showed that cancer incidence rose with age, but not with diabetes duration, suggesting that metabolic disturbances, including adiposity and insulin resistance, are probably associated with oncogenic processes before the onset of type 2 diabetes [21]. However, hyperglycaemia itself is implicated, as evidenced by the elevated cancer risk in type 1 diabetes [22, 23], which is less associated with insulin resistance. Additionally, consistent associations have been reported between HbA_1c_ and cancer incidence in type 2 diabetes, independent of insulin resistance or adiposity markers [6, 8, 17, 24–27]. Our findings confirm the positive association between HbA_1c_ and incident cancer independent of the triglyceride-to-HDL-cholesterol ratio and BMI, which were surrogates for insulin resistance and adiposity, respectively.

Expanding on this evidence, our study provides new insights suggesting that the association between HbA_1c_ values and incident cancer at any site becomes noticeable within the first 2 years of diabetes diagnosis at HbA_1c_ values ≥53 mmol/mol (7.0%). Not surprisingly, prolonged exposure to high HbA_1c_ values was associated with increasing risk of incident cancer. These findings extend previous observations on the possible enduring patterns related to early glycaemic exposure, and indicate that suboptimal glycaemic management soon after diabetes diagnosis may be associated with higher subsequent risks of cancer at any site, in addition to vascular complications [11].

Previous studies have suggested that the risk of diabetes-related complications is directly proportional to cumulative glycaemic burden, i.e. mean HbA_1c_ values and duration of exposure, regardless of when hyperglycaemia occurs [28]. However, UKPDS data revealed that historical HbA_1c_ contributions to all-cause mortality and myocardial infarction risk varied temporally [19]. Using a WCE model, which enables a more flexible representation of the risk contribution of past HbA_1c_ values, our findings suggest that the timing of hyperglycaemic exposure modifies the cancer risk. Specifically, early hyperglycaemia (within the initial years after diagnosis) was associated with a disproportionately higher cancer risk than later exposure. The time-varying risk weighted for cancer mirrored the declining trend seen for myocardial infarction in both our validation analysis and the UKPDS data [19], albeit with smaller magnitudes. Importantly, we showed that, even with an equivalent cumulative glycaemic burden, individuals with better early glycaemic management had a lower cancer risk than those whose control improved later. These findings could have clinical implications, and suggest the need for strategies that prioritise early glycaemic management in people with type 2 diabetes.

Hyperglycaemia, particularly when experienced soon after diagnosis of diabetes, may be linked to cancer through the following biological pathways. First, formation of advanced glycation end-products induced by hyperglycaemia promotes the formation of reactive oxygen species, which have been found to stimulate tumour cell growth, invasion and migration [29, 30]. Early exposure to hyperglycaemia may lead to the early formation and accumulation of advanced glycation end-products. Second, hyperglycaemia activates the renin–angiotensin–aldosterone system (RAAS) [31, 32], and the increased interaction between angiotensin II and angiotensin II type 1 receptors has been shown to enhance cancer cell proliferation and angiogenesis [33]. Additionally, evidence has suggested crosstalk between the RAAS and lipid metabolism, whereby hyperglycaemia-induced oxidation of LDL-cholesterol further enhances the expression of RAAS components to increase cancer risk [34–37]. Third, cancer cells consume more glucose as an energy source than non-transformed cells, and hyperglycaemia may thus confer a growth advantage. However, in vitro studies indicate that increasing glucose concentrations beyond 5 mmol/l does not further enhance tumour proliferation, despite glucose depletion slowing growth [38]. Lastly, early hyperglycaemia may be associated with lasting epigenetic changes, such as modifications to histones and DNA [39], potentially related to irreversible alterations in chromatin structure and the miRNA signature. These changes could accumulate and alter gene expression patterns in a way that favours tumorigenesis [40, 41].

Several limitations warrant consideration. First, our study population consisted exclusively of individuals who use public health services, who potentially differ in terms of demographic characteristics, socioeconomic status and prognosis from those receiving private healthcare. However, private healthcare services account for only about 10% of the overall healthcare provision in Hong Kong [42]. Second, our findings must be interpreted in the light of potential misclassification due to diabetes that may have existed but remained undiagnosed before study entry. Third, conditions that may affect the accuracy of HbA_1c_ measurements, such as haemoglobinopathies, cirrhosis or severe anaemia, may introduce measurement error for a small subset of individuals, potentially biasing our estimates towards the null. However, the prevalence of these conditions is low (<1%) [43–45]. Fourth, the shorter follow-up duration observed in the highest-risk glycaemic group may reflect both a higher incidence of early adverse events and poorer healthcare engagement. This creates a potential bias, as the factors leading to shorter follow-up, such as infrequent clinic attendance, are themselves likely to be associated with lifestyle and behavioural cancer risk factors for which we could not fully adjust. Fifth, residual confounding remains possible due to unmeasured cancer-specific risk factors, including menopausal status, use of hormone replacement therapy, family history of cancer, lifestyle and cancer screening history. Sixth, the observed association between early high HbA_1c_ exposure and cancer risk may reflect the impact of undiagnosed cancer on HbA_1c_ rather than being a true ‘legacy effect’ of glycaemic exposure. However, we performed a sensitivity analysis that excluded cancer cases within 5 years of diagnosis, and the findings in the primary analysis remained. Seventh, potential loss to follow-up due to external migration is a concern. However, considering that the net migration rate in Hong Kong from 2001 to 2019 was less than 1% [46], the impact on our findings is probably minimal. Eighth, the outcome events were identified using diagnosis codes during hospitalisation, which may have led to under-reporting of cancer cases in individuals who were not hospitalised for their condition. Ninth, although sex was adjusted for in the analyses, we did not perform formal sex-specific analyses. Therefore, potential differences in the association across sexes could not be fully evaluated, which may limit interpretation of sex-specific effects and the generalisability of the findings across sexes. Last, given the retrospective nature of our study, our results cannot be considered to establish causality.

### Conclusions

In conclusion, we confirmed the epidemiological association between hyperglycaemia and incident cancer in people with type 2 diabetes. We further showed that hyperglycaemia in the first 5 years of diabetes diagnosis may be associated with cancer risks in the longer term, irrespective of the glycaemic burden in the subsequent period. Notably, early exposure may be associated with a greater increase in cancer incidence compared with later hyperglycaemia. These findings support the possible benefit of early and sustained reduction of blood glucose in people with newly diagnosed type 2 diabetes in terms of their long-term risk of cancer.

## Supplementary Information

Below is the link to the electronic supplementary material.

ESM (PDF 625 KB)

## Data Availability

The data underlying the results presented in the study are hosted by the Hong Kong Hospital Authority. Due to local regulations, the data are not available to the public. Requests for data access may be submitted via the Hong Kong Hospital Authority data-sharing portal, and will be considered on a case-by-case basis for research purposes, subject to institutional approval and relevant ethical and regulatory requirements. Data will be made available only to eligible researchers through the Hospital Authority’s formal application mechanism and may be subject to restrictions on use. Further information is available at https://www3.ha.org.hk/data.

## Abbreviations

DPP-4: Dipeptidyl peptidase 4
RAAS: Renin–angiotensin–aldosterone system
WCE: Weighted cumulative exposure

## Author relationships and activities

AOYL has served as a member of advisory panels for Amgen, AstraZeneca, Boehringer Ingelheim and Sanofi, and has received research support from Amgen, the Asia Diabetes Foundation, Bayer, Boehringer Ingelheim, Lee’s Pharmaceutical, MSD, Novo Nordisk, Roche, Sanofi, Sugardown Ltd and Takeda, outside the submitted work. APSK has received research grants and/or speaker honoraria from Abbott, Astra Zeneca, Bayer, Boehringer Ingelheim, Eli Lilly, Merck Serono, Nestle, Novo Nordisk, Pfizer and Sanofi, outside the submitted work. JCNC has received grants from Astra Zeneca, Lilly, Hua Medicine and Lee Powder, and grants and personal fees from Bayer, MSD, Merck and Sanofi, acts as the CEO of the Asia Diabetes Foundation and is a founding director of GemVCare, outside the submitted work. In addition, she has obtained a patent on genetic markers for diabetic kidney disease. RCWM is a member of the editorial board of *Diabetologia*, and has received grants from AstraZeneca, Bayer, Boehringer Ingelheim, Novo Nordisk and Roche Diagnostics (HK) Limited, and speaker honoraria or consultancy fees from AstraZeneca, Bayer, Boehringer Ingelheim, Eli Lilly, Medtronic, Merck, Novo Nordisk and Roche Diagnostics. All speaker honoraria or consultancy fees have been donated to support diabetes research. RCWM also reports support from the Hong Kong Government Innovation and Technology Commission and its Technology Start-Up Support Scheme for Universities (TSSSU), as co-founder of the technology start-up GemVCare, which provides genetic testing for diabetes and diabetes complications, outside the submitted work. The remaining authors declare that there are no relationships or activities that might bias, or be perceived to bias, their work.
