# Supplementary material for: Early glycaemic exposure and cancer risk in people with newly diagnosed type 2 diabetes
Source: Diabetologia. 2026 Jun 5;69(9):2512–22. doi: 10.1007/s00125-026-06758-7 (PMC13424208; doi:10.1007/s00125-026-06758-7)

ESM Methods

Detailed descriptions on inclusion criteria

As shown in ESM Fig. 1, we excluded people with type 1 diabetes or unknown diabetes type, or those with a history of cancer. To ensure reliable assessment of glycaemic exposure, we excluded individuals with fewer than two HbA_{1c} measurements during the entire observation period for Aim 1, or during each early exposure period for Aim 2. To mitigate the risk of reverse causality (i.e., undiagnosed preclinical cancer influencing glycaemic control rather than vice versa), we applied a 2-year exclusion window before the cancer diagnosis. Specifically, individuals who developed cancer within 2 years of their diabetes diagnosis were excluded. For those who were followed for more than 2 years before developing cancer, any HbA_{1c} values measured within 2 years before cancer were also excluded. This 2-year period was selected based on prior studies indicating that it is sufficiently long to minimize the confounding effects of subclinical malignancy on HbA_{1c} [1].

Multiple imputation for missing laboratory values

Missing laboratory measurements of HbA_{1c}, systolic and diastolic blood pressures, body mass index (BMI), triglyceride-to-high-density lipoprotein (HDL) ratio, and low-density lipoprotein (LDL)-cholesterol values were imputed using multiple imputation by chained equations (MICE) implemented in R (mice package, version 3.19.0). The imputation model included all analysis variables, with the predictor matrix automatically specified using the quickpred() function. Continuous variables were imputed using predictive mean matching, and binary variables using logistic regression. We generated 20 imputed datasets with 20 iterations per chain after a burn-in period of 10 iterations. Convergence was assessed by inspecting trace plots of imputed values. Analyses were performed separately on each imputed dataset, and results were combined using Rubin's rules [2] via the pool() function in R.

Detailed descriptions on the weighted cumulative exposure model

The weighted cumulative exposure (WCE) model estimated a weighting function which quantifies the contribution of historical glycaemic exposures at different time points to cancer risk. This function allows us to identify which periods of historical HbA_{1c} exposure had a greater influence on cancer risk, with higher weights indicating a greater impact of exposure during those periods. To improve interpretability and avoid potential misinterpretation due to sparse data at the extremes of the follow-up period, we restricted the weighting function to a time window capped at the 95th percentile of follow-up duration. To compare cancer risk between two theoretical HbA_{1c} profiles, we calculated the weighted cumulative exposure by multiplying the estimated weighting function to the respective HbA_{1c} time-series profiles. The WCE model was then used to estimate the HRs for the two profiles, with CIs computed using 500 bootstrap iterations. As validation of this method, we compared the predictive performance of the WCE model with that of a conventional Cox proportional hazards model, where HbA_{1c} was modelled as a time-varying covariate. This comparison was performed using the log-likelihood statistics and area under curve (AUC) at 10 years after the diagnosis of type 2 diabetes. Additionally, we repeated the analysis for gastrointestinal (GI) bleeding and myocardial infarction (MI) as negative and positive controls [3], respectively. GI bleeding was chosen as a negative control because it was not expected to be directly influenced by glycaemic exposure, whereas MI was chosen as a positive control due to its established association with glycaemic levels, as well as the demonstrated legacy effect for MI in a previous study [4].

The mathematical formulation of the WCE model is adapted from Sylvestre and Abrahamowicz (2009) [5], reproduced here for completeness. The WCE metric captures the cumulative impact of historical exposures at a given time u during follow-up. It is expressed as:

$$\mathrm{WCE}(u) = \sum_{t}^{u} w(u-t)X(t) \quad (1)$$

Where $X(t)$ represents exposure level at time t, $t \leq u$ represents all past exposure times up to the current time u, and $w(u-t)$ is the weighting function, which quantifies the contribution of each past exposure depending on how much time has elapsed since it occurred. This WCE value is used as a time-dependent covariate in the proportional hazards Cox model:

$$h(u \mid X(u), Z(u)) = h_0(u) exp\left[\beta \sum_t^u w(u-t)X(t) + \sum_{s=1}^q \eta_s Z_s(u)\right] \quad (2)$$

In this equation, $h_0(u)$ represents the baseline hazard function, while $X(u) = \{X(t), 0 \leq t \leq u\}$. The $Z_s(u)$ are other covariates. In practical applications, the exact form of the weighting function $w(u-t)$ is typically unknown and must be estimated, subject only to general smoothness conditions. The $w(u-t)$ is estimated using cubic B-splines, a flexible method allowing the weighting function to vary based on the exposure history:

$$w(u-t) = \sum_{j=1}^m \theta_j B_j(u-t) \quad (3)$$

where $B_j,\ j = 1, \dots, m$ are the cubic spline basis functions, and $\theta_j,\ j = 1, \dots, m$ are their corresponding coefficients. This approach allows for an adaptable estimation of the weights that reflect how different historical exposures affect the risk. Both β from equation (2) and θ_j from equation (3) measure the relationship between exposure and risk. To avoid identifiability issues when estimating these parameters simultaneously, the WCE model redefine:

$$\gamma_j = \beta\theta_j,\ j = 1, \dots, m \quad (4)$$

To simplify the estimation process, the artificial time-dependent covariates are introduced:

$$D_j(u) = \sum_t^u B_j(u-t)X(t),\ j = 1, \dots, m \quad (5)$$

By introducing these artificial covariates, the Cox model can be reformulated:

$$h(u \mid X(u), Z(u)) = h_0(u) exp\left[\sum_{j=1}^m \gamma_j D_j(u) + \sum_{s=1}^q \eta_s Z_s(u)\right] \quad (6)$$

With this reformulation, the parameters can be estimated using any standard software capable of handling Cox regression with time-dependent covariates. The hazard ratios (HRs) comparing individuals with different exposure vectors $X_1(t)$ and $X_0(t),\ t \leq u$, at any time u during follow-up can be calculated as:

$$exp\left[\sum_{j=1}^m \hat{\gamma}_j \sum_t^u B_j(u-t)[X_1(t) - X_0(t)]\right] \quad (7)$$

This approach allows for estimating HRs in various scenarios, such as altering exposure intensity, adjusting the timing of exposure, or combining different exposure changes. Confidence intervals for these HRs can be obtained using bootstrap resampling.

[1] Lemanska A, Price CA, Jeffreys N, et al. (2022) BMI and HbA1c are metabolic markers for pancreatic cancer: Matched case-control study using a UK primary care database. PLOS ONE 17(10): e0275369. 10.1371/journal.pone.0275369

[2] Royston P (2004) Multiple imputation of missing values. The Stata Journal 4(3): 227-241

[3] Lipsitch M, Tchetgen Tchetgen E, Cohen T (2010) Negative controls: a tool for detecting confounding and bias in observational studies. Epidemiology 21(3): 383-388. 10.1097/EDE.0b013e3181d61eeb

[4] Lind M, Imberg H, Coleman RL, Nerman O, Holman RR (2021) Historical HbA(1c) Values May Explain the Type 2 Diabetes Legacy Effect: UKPDS 88. Diabetes Care 44(10): 2231-2237. 10.2337/dc20-2439

[5] Sylvestre MP, Abrahamowicz M (2009) Flexible modeling of the cumulative effects of time-dependent exposures on the hazard. Stat Med 28(27): 3437-3453. 10.1002/sim.3701

ESM Table 1. Definitions of cancers.

	Classification	ICD-9	ICD-10
All-site cancer		140:208	C00:C97
Malignant neoplasm of thyroid	Diabetes-related and Obesity-related	193	C73
Malignant neoplasm of oesophagus	Diabetes-related and Obesity-related	150	C15
Malignant neoplasm of pancreas	Diabetes-related and Obesity-related	157	C25
Malignant neoplasm of liver and intrahepatic bile ducts	Diabetes-related and Obesity-related	155	C22
Malignant neoplasm of colon, rectosigmoid junction, and rectum (i.e., colorectal)	Diabetes-related and Obesity-related	153, 154	C18, C19, C20
Malignant neoplasm of gallbladder	Diabetes-related and Obesity-related	156.0	C23
Malignant neoplasm of breast (postmenopausal)	Diabetes-related and Obesity-related	174-175	C50
Malignant neoplasm of corpus uteri (endometrium)	Diabetes-related and Obesity-related	182	C54
Malignant neoplasm of ovary	Diabetes-related and Obesity-related	183.0	C56
Malignant neoplasm of kidney	Diabetes-related and Obesity-related	189.0	C64
Malignant neoplasm of bladder	Diabetes-related cancers	188	C67
Non-Hodgkin lymphoma	Diabetes-related cancers	200-202	C82-C85
Leukaemia	Diabetes-related cancers	204-208	C91-C95
Multiple myeloma	Obesity-related cancers	203	C90.0

ESM Table 2. Proportion of participants with at least one missing laboratory measurement during follow-up (overall and at 2, 4, 6, 8 years post-diagnosis) for Aim 3, stratified by the mean HbA_{1c} values within the first two years after diabetes diagnosis.

	<7.0%	7.0%-7.9%	8.0%-8.9%	≥9.0%
Number	17,426	11,927	6,923	13,690
Overall				
HbA_{1c}	16.68%	15.34%	16.54%	17.70%
BMI	32.84%	31.58%	32.62%	35.03%
Systolic blood pressure	32.46%	31.16%	32.15%	34.75%
Diastolic blood pressure	32.73%	31.44%	32.31%	34.63%
LDL cholesterol	37.47%	35.35%	36.19%	38.47%
Triglyceride-to-HDL-cholesterol ratio	37.74%	35.83%	36.70%	39.20%
2 years				
HbA_{1c}	0.00%	0.00%	0.00%	0.00%
BMI	4.11%	3.97%	4.16%	4.95%
Systolic blood pressure	3.51%	3.61%	3.55%	4.82%
Diastolic blood pressure	3.87%	3.76%	3.71%	4.27%
LDL cholesterol	13.35%	11.91%	10.89%	11.76%
Triglyceride-to-HDL-cholesterol ratio	11.88%	10.29%	8.83%	8.61%
4 years				
HbA_{1c}	11.07%	10.10%	10.77%	11.38%
BMI	18.75%	18.07%	18.61%	20.67%
Systolic blood pressure	18.28%	17.60%	18.08%	20.39%
Diastolic blood pressure	18.52%	17.85%	18.27%	20.12%
LDL cholesterol	25.86%	24.19%	24.09%	26.10%
Triglyceride-to-HDL-cholesterol ratio	24.89%	23.03%	22.63%	24.24%
6 years				
HbA_{1c}	15.34%	13.94%	14.88%	15.84%
BMI	25.14%	24.01%	24.73%	27.25%
Systolic blood pressure	24.70%	23.50%	24.20%	26.99%
Diastolic blood pressure	24.99%	23.78%	24.42%	26.84%
LDL cholesterol	31.04%	29.11%	29.63%	32.09%
Triglyceride-to-HDL-cholesterol ratio	30.19%	28.08%	28.43%	30.65%
8 years				
HbA_{1c}	16.76%	15.41%	16.28%	17.31%
BMI	28.76%	27.54%	28.18%	30.89%
Systolic blood pressure	28.34%	27.06%	27.68%	30.63%
Diastolic blood pressure	28.62%	27.36%	27.89%	30.52%
LDL cholesterol	34.05%	32.21%	32.63%	35.47%
Triglyceride-to-HDL-cholesterol ratio	33.24%	31.28%	31.60%	34.23%

ESM Fig. 1. Flow chart of the study design.

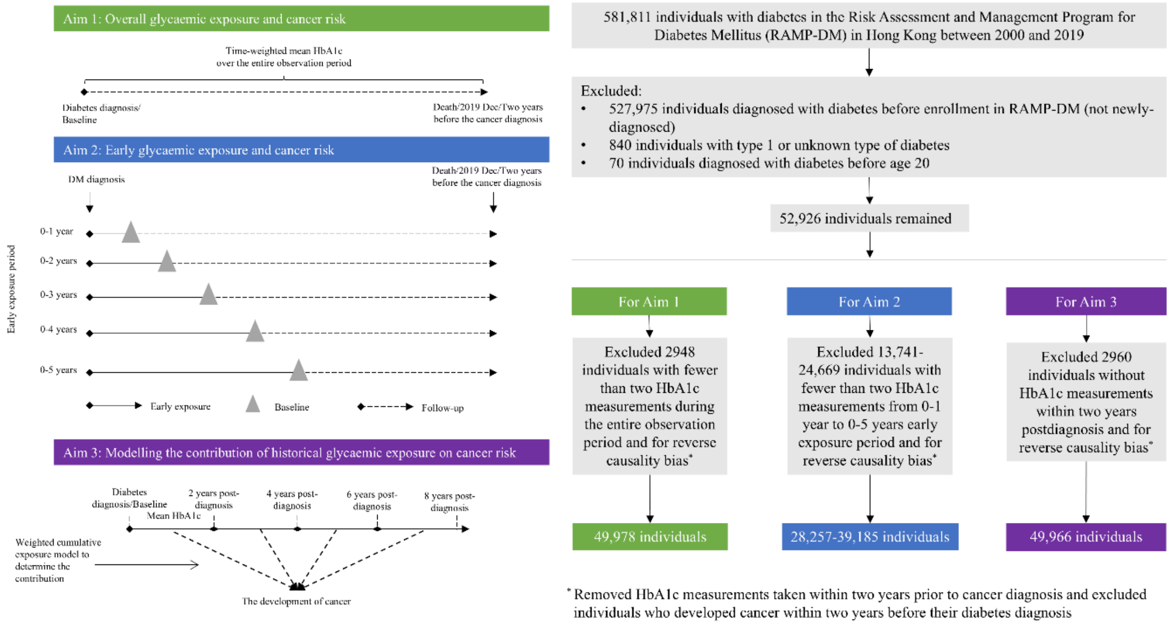

ESM Fig. 2. Sensitivity analysis of extending the exclusion period for cancer cases occurring after the T2DM diagnosis from 2 years to 5 years. A: Forest plots of the association between attained HbA_{1c} during each early exposure period and incident hazards of all-site cancer, with HbA_{1c} <53 mmol/mol (<7%) as the reference group. B: Weight functions of historical HbA_{1c} values for incident risks of all-site cancer. C: Hazard ratios for incident hazards of all-site cancer, following a 11 mmol/mol (1%) HbA_{1c} reduction implemented at different periods over a 10-year window post-T2D diagnosis. Models were adjusted for age at diabetes diagnosis, sex, calendar year at diagnosis, smoking, drinking, systolic and diastolic blood pressure, body mass index, triglyceride-to-HDL-cholesterol ratio, LDL cholesterol, use of oral glucose-lowering drugs (metformin, sulphonylurea, DPP-4 inhibitors, thiazolidinediones, and others) and insulin, and histories of cardiovascular disease, congestive heart failure, and chronic kidney disease.

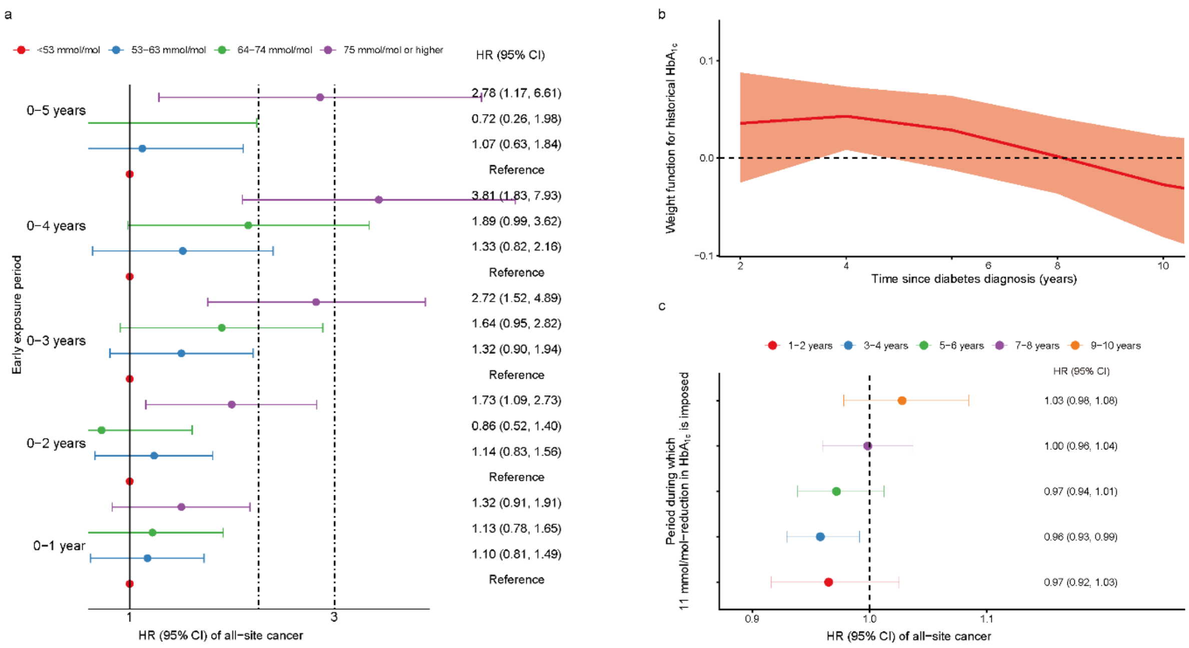

ESM Fig. 3. Differences in Log-Likelihood statistics and 10-year AUCs between the WCE model and conventional Cox model. Models were adjusted for age at diabetes diagnosis, sex, calendar year at diagnosis, smoking, drinking, systolic and diastolic blood pressure, body mass index, triglyceride-to-HDL-cholesterol ratio, LDL cholesterol, use of oral glucose-lowering drugs (metformin, sulphonylurea, DPP-4 inhibitors, thiazolidinediones, and others) and insulin, and histories of cardiovascular disease, congestive heart failure, and chronic kidney disease.

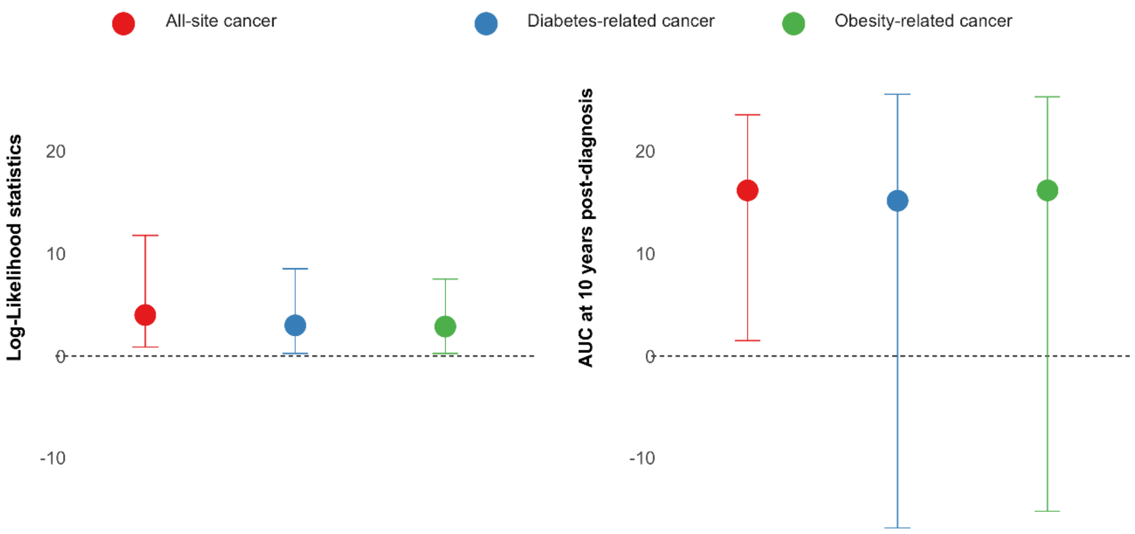

ESM Fig. 4. Weight functions of historical HbA_{1c} values for incident hazards of myocardial infarction and gastrointestinal bleeding. A higher weight function means a greater contribution of historical exposure at that time point to the outcome risk. Models were adjusted for age at diabetes diagnosis, sex, calendar year at diagnosis, smoking, drinking, systolic and diastolic blood pressure, body mass index, triglyceride-to-HDL-cholesterol ratio, LDL cholesterol, use of oral glucose-lowering drugs (metformin, sulphonylurea, DPP-4 inhibitors, thiazolidinediones, and others) and insulin, and histories of cardiovascular disease, congestive heart failure, and chronic kidney disease.

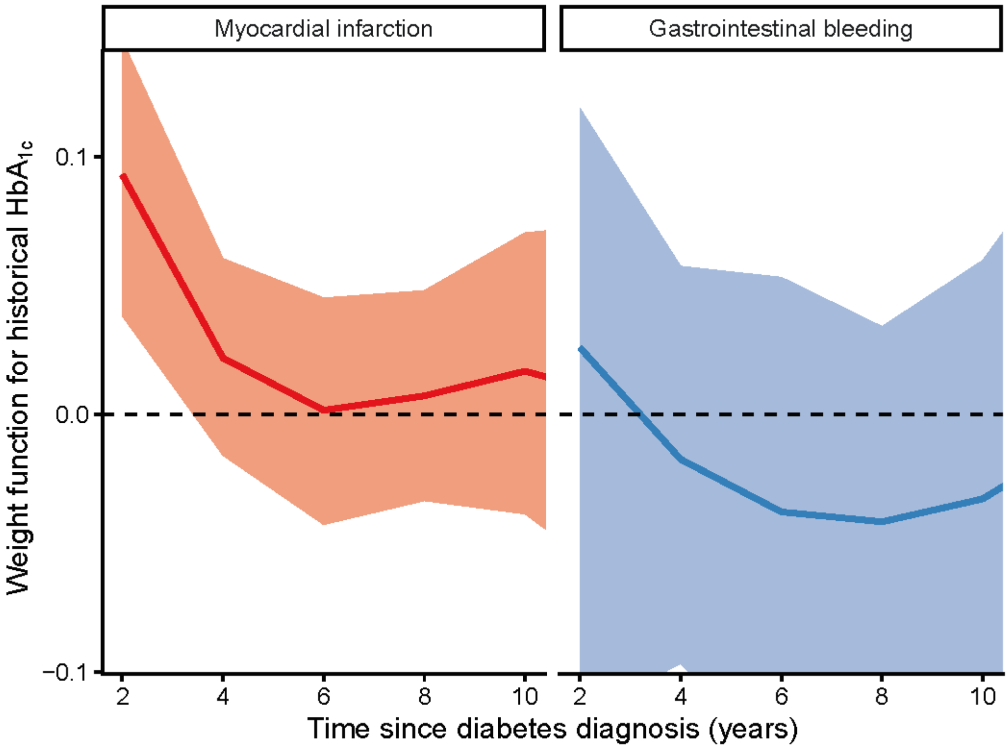

ESM Fig. 5. Hazard ratios for incident hazards of myocardial infarction (a) and gastrointestinal bleeding (b), following a 1% HbA_{1c} reduction implemented at different periods over a 10-year window post-T2D diagnosis. Models were adjusted for age at diabetes diagnosis, sex, calendar year at diagnosis, smoking, drinking, systolic and diastolic blood pressure, body mass index, triglyceride-to-HDL-cholesterol ratio, LDL cholesterol, use of oral glucose-lowering drugs (metformin, sulphonylurea, DPP-4 inhibitors, thiazolidinediones, and others) and insulin, and histories of cardiovascular disease, congestive heart failure, and chronic kidney disease.

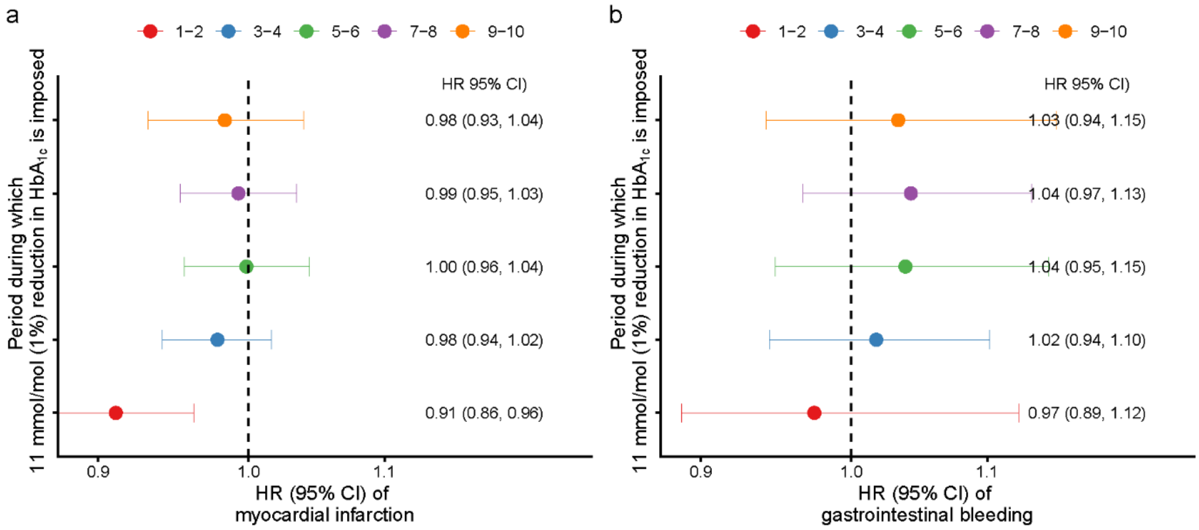

Supplement: Supplementary file 1 — ESM (PDF 625 KB) [file 125_2026_6758_MOESM1_ESM.pdf]
